# Investigation of the transcriptional impact of rare germline JAK/STAT variants found in a Tyrolean alpine community

**DOI:** 10.1186/s12864-025-12307-0

**Published:** 2025-12-01

**Authors:** Lothar Hennighausen, Teemu Haikarainen, Sung-Gwon Lee, Yasemin Caf, Priscilla A. Furth, Olli Silvennoinen, Hye Kyung Lee, Ludwig Knabl

**Affiliations:** 1 National Institute of Diabetes, Digestive and Kidney Diseases, National Institutes of Health, Bethesda, MD 20892 USA; 2 Faculty of Medicine and Health Technology, Tampere University, Tampere, Finland; 3 Fimlab Laboratories, Tampere, Finland; 4 Institute of Biotechnology, HiLIiFE, University of Helsinki, Helsinki, Finland; 5 Y2L2Science GmbH, Hauptplatz 4, Zams, 6511 Austria

**Keywords:** JAK/STAT genes, Missense variants, *In Silico* prediction tools, Immune regulatory genes, AlphaFold predicted protein structure

## Abstract

The impact of most missense variants in immune regulatory genes on steady-state immune transcriptome expression in healthy individuals in real-world conditions remains largely unexplored. Here, we investigated the transcriptional impact of 21 germline variants in *JAK*, *TYK2*, and *STAT* family genes identified within a healthy Austrian alpine cohort. Of the 98 participants 35 carried only one variant and 32 carried two or more variants. Allele frequencies (AF) of these germline mutations, based on the gnomAD and All of Us databases, ranged from approximately 10^− 6^ for three very rare variants to approximately 10^− 1^ for the most common. In silico prediction tools combined with surveying COSMIC and the NIH ClinVar databases were used to explore links with known disease. Thirteen of the 21 variants were reported in the COSMIC database, suggesting gain-of-function activity. Structural analysis revealed clear mechanistic basis for few variants, although some appeared to have modest structural effects in agreement with their weak GOF phenotypes.

**Supplementary Information:**

The online version contains supplementary material available at 10.1186/s12864-025-12307-0.

## Background

Janus Kinases (JAKs) and Signal Transducers and Activators of Transcription (STATs) play indispensable roles in translating cytokine signals into transcriptional responses that regulate a range of biological processes, including body growth [1], lactation [2] and hematopoiesis [2–4]. Missense variants are present in over 50% of the codons within the four JAK family members (JAK1, JAK2, JAK3, and TYK2) and in the seven STAT proteins (STAT1, STAT2, STAT3, STAT4, STAT5A, STAT5B and STAT6) [5]. While some variants, such as JAK2^V617F^ [6, 7], are well-characterized in their association with clinical disease, the biological significance of most variants remains unclear. Notably, the biological impact of germline and somatic variants could be restricted to specific cell types or unique physiological circumstances, such pregnancy-induced lactation or cytokine triggered immune responses.

In a previous study we investigated the immune activation following asymptomatic SARS-CoV-2 infection through a comparative investigation of the immune cell transcriptomes in asymptomatic seropositive and highly exposed seronegative individuals from the same community 4–6 weeks after a superspreading event [8]. In summary, whole blood transcriptomes identified individual immune profiles within a community population and showed that asymptomatic infection within a COVID-19 super-spreading event was not associated with enduring general immunological activation. However, it was not clear whether the sustained transcriptome differences between individuals in this community were due to underlying variants in the innate or adaptive immune system. To further investigate the possibility that variants in the JAK/STAT signaling pathway modulate the regulation of immune transcriptome, we analyzed JAK/STAT variants in this cohort of 95 individuals from the Ischgl community and associated the genetic information with transcriptome profiles from peripheral immune cells.

## Materials and methods

### Cohort description

DNA and RNA analyses were performed on *n* = 98 individuals from the same alpine community that were originally studied for their transcriptional response to past COVID infection [8]. For detailed information see Supplementary Tables 1 and 2 (Tables S1 and S2). *n* = 67 subjects were identified that had JAK/STAT mutations. The total cohort investigated for the transcriptional impact of rare germline JAK/STAT variants included *n* = 57 males (age mean +/− standard deviation (SD) 37 +/− 21 years, range 3–86 years) and *n* = 41 females (37 +/− 19 years, range 1–65 years). Those who had JAK/STAT mutations included *n* = 39 males (38 +/− 22 years, range 3–86 years). and *n* = 28 females (38 +/− 19 years, range 1–63 years). All individuals were in a state of self-described stable health at the time samples for RNA sequencing were drawn. Four to six weeks prior to RNA sample acquisition the community experienced a wave of pandemic wild-type virus (D614G) and B.1.1.7 variant SARS-CoV-2 infection [8, 9]. *N* = 42 individuals within the cohort experienced asymptomatic SARS-CoV-2 infection and *n* = 56 individuals were highly exposed but were asymptomatic and did not develop an antibody response to SARS-CoV-2 [8]. Within this study there was no significant difference in the frequency of JAK-STAT SNV prevalence between these two groups (67% of the individuals that experienced an asymptomatic SARS-CoV-2 infection were found to carry a JAK/STAT SNV and 70% of the highly-exposed seronegative were found to carry a JAK/STAT SNV). Health records of the entire cohort were reviewed for diagnoses of health conditions related to immunological disease, cancer and allergy. Nine individuals within the cohort were identified with possible immunologically-related disease (Table S2). All carried a JAK/STAT SNV: asthma (*n* = 1, Stat2^S23L^, TYK2^V362F^), chronic sinusitis (*n* = 1, STAT2^Q826H^, TYK2^G363S^), Hashimoto’s thyroiditis (*n* = 1, TYK2^G363S^), hypothyroid (*n* = 1, STAT2^Q826H^, *n* = 1 TYK2^V362F, A928V^), subacute thyroiditis (*n* = 1, STAT2^M594I^), increased anti-nuclear antibody (ANA) of unknown origin (*n* = 1, no JAK/STAT SNV), Crohn’s disease (*n* = 1, no JAK/STAT SNV), rheumatism (*n* = 1, TYK2^V362F, I684S^). Four individuals within the cohort reported a cancer diagnosis: acute lymphocytic leukemia (*n* = 1, no JAK/STAT SNV), gastric cancer (*n* = 1, STAT2^G825C^), multiple endocrine neoplasia (MEN) 3 (*n* = 1, STAT2^Q826H^), breast microcarcinoma (*n* = 1, TYK2^V362F, I864S, A928V^). Four individuals reported penicillin allergy (*n* = 1, STAT2^M594I^, *n* = 1 STAT2^S23L^, TYK2^V362F^, *n* = 1 STAT2^S23L^, *n* = 1 TYK2^V362F, I684S^). Eighteen individuals reported grass allergy (*n* = 5, no JAK/STAT SNV, *n* = 3 TYK2^V362F^, *n* = 1 STAT2^Q826H^, TYK2^V690L^, *n* = 2 STAT2^M594I^, *n* = 1 STAT2^M594I^, TYK2^V362F^, *n* = 1 TYK2^V362F, A298V^, *n* = 1 JAK2^L393V^, *n* = 1 STAT2^G825C^, *n* = 1 TYK2^G363S^, *n* = 1 TYK2^V362F, I884S^, *n* = 1, TYK2^V362F, I864S, A928V^. Three individuals reported allergy to animal skin/hair: *n* = 1, no JAK/STAT SNV, *n* = 1 TYK2^V362F^, *n* = 1 TYK2^V362F, A928V^. One female individual included in the study had Nuclear factor-kappa B Essential Modulator (NEMO) deficiency (IKBKG exon4_10del mutation). She also reported a bee, wasp, hornet al.lergy (JAK3^V722I^, TYK2^I864S, A928V^). One subject from the original study was an individual with an underlying diagnosis of cystic fibrosis (CF) who carried four variants, the activating JAK2^R1063H^ variant, STAT2^M594I^, TYK2^V362F^ and TYK2^I684S^, but due to the underlying diagnosis of CF the transcriptome of this individual was not included in the analysis.

### Whole genome sequencing and data analysis

Genomic DNA was extracted from human buffy coat samples using the Wizard Genomic DNA Purification Kit (Promega). PCR-free library preparation and whole-genome sequencing were performed at the NIH Intramural Sequencing Center (NISC). In this process, PCR-free libraries were constructed from 1 µg of genomic DNA utilizing the TruSeq DNA PCR-Free HT Sample Preparation Kit (Illumina). The median fragment size of the libraries was approximately 400 base pairs (bp). Each library was tagged with unique dual-index DNA barcodes to facilitate library pooling while minimizing the occurrence of barcode hopping. Libraries were pooled and sequenced on the NovaSeq 6000 or NovaSeqX+ (Illumina) platforms, generating a minimum of 300 million paired-end reads (151 base pairs each) per individual library.

Subsequent data analysis was conducted using the Clara Parabricks Pipeline (version 4.0.1) for the variants’ detection (https://docs.nvidia.com/clara/parabricks/4.0.1/whatsnew.html). Alignment of the sequencing reads was performed with BWA MEM [10] using the human reference genome (hg38), followed by duplicate marking with Picard tools [11]. The Genome Analysis Toolkit (GATK) pipeline was employed for base quality score recalibration (BQSR) and variant calling, with HaplotypeCaller generating gvcf files using a minimum Phred-scaled confidence threshold of 16.

### Single nucleotide variant (SNV) detection in RNA-seq data

The raw data generated from RNA of human buffy coat were subjected to QC analyses using the FastQC tool (version 0.11.9) (https://www.bioinformatics.babraham.ac.uk/projects/fastqc/). mRNA-seq read quality control was done using Trimmomatic [12] (version 0.36) and STAR 2-pass procedure [13] (version STAR 2.7.9a) was used to align the reads (hg19). Aligned reads were filtered using BWA MEM [10] (version 0.7.15), followed by Picard tools [11] (version 2.9.2) to mark duplicates. The GATK analysis workflow was applied as follows: (i) base recalibration - GATK BaseRecalibrator, AnalyzeCovariates, and PrintReads - using the databases of known polymorphic sites, dbSNP138 (provided by the high-performance computing team of the NIH (Biowulf)); (ii) variant calling - GATK HaplotypeCaller - with the genotyping mode “discovery”, the “ERC” parameter for creating gvcf and a minimum phred-scaled confidence threshold of 30. Hard filters were applied: QD < 2.0 || QUAL < 30.0 || SOR >3.0 || FS >60.0 || MQ < 40.0 || MQRankSum < −12.5 || ReadPosRankSum < −8.0. The resulting SNVs were additionally filtered by removing those overlapping with repetitive elements [14] (UCSC’s masked repeats plus simple repeats; https://hgdownload.soe.ucsc.edu/goldenPath/hg19/database/) and black regions (ENCODE [15]; https://mitra.stanford.edu/kundaje/akundaje/release/blacklists/hg19-human/). On an individual level, only SNVs with a genotype of 0/1 or 1/1 were kept. Further filtering steps comprised the removal of SNVs with a read depth smaller than 10, an excessive read depth [16] (d + 3√d, d = average read depth), an allele frequency less than 10% using a variety of tools (BEDtools, version 2.26.0; BEDOPS, version 2.4.3; VCFtools, version 0.1.17) [17–19]. All SNVs within +/- 5 bp of an indel border were removed as likely false-positives. SNP position on hg19 was converted to hg38.

### mRNA sequencing (mRNA-seq) data analysis

The raw data were subjected to QC analyses using the FastQC tool (version 0.11.9). mRNA-seq read quality control was done using Trimmomatic [12] (version 0.36) and STAR RNA-seq [13] (version STAR 2.7.9a) using 150 bp paired-end mode was used to align the reads (hg19). HTSeq [20] (version 0.9.1) was to retrieve the raw counts and subsequently, Bioconductor package DESeq2 [21] in R (https://www.R-project.org/) was used to normalize the counts across samples [22] and perform differential expression gene analysis. Additionally, the RUVSeq [23] package was applied to remove confounding factors. The data were pre-filtered keeping only genes with at least ten reads in total. The visualization was done using dplyr (https://CRAN.R-project.org/package=dplyr) and ggplot2 [24]. P-values were calculated using a paired, two-side Wilcoxon test and adjusted p-value (pAdj) corrected using the Benjamini–Hochberg method. The cut-off value for the false discovery rate was pAdj >0.05. Genes with log_2_ fold change >1 or <−1, pAdj < 0.05 and without 0 value from all sample were considered significant and then conducted gene enrichment analysis (GSEA, https://www.gsea-msigdb.org/gsea/msigdb). For significance of each GSEA category, significantly regulated gene sets were evaluated with the Kolmogorov-Smirnov statistic. A value of *P < 0.05, **P < 0.01, ***P < 0.001, ****P < 0.0001 was considered statistically significant.

### Allele frequency information and in silico prediction of altered function

Allele frequency information for certain variants was collected using multiple databases, like dbSNP (https://www.ncbi.nlm.nih.gov/snp/), gnomAD (https://gnomAD.broadinstitute.org/), All of Us (https://workbench.researchallofus.org), COSMIC (https://cancer.sanger.ac.uk/cosmic) and ClinVar (https://www.ncbi.nlm.nih.gov/clinvar/).

Multiple computational tools were employed to assess the potential for altered function of the JAK/STAT variants. AlphaMissense [25] (https://alphamissense.hegelab.org/search) was used to predict the functional impact of variants, with scores ranging from 0 (benign) to 1 (pathogenic). The Rare Exome Variant Ensemble Learner (REVEL) [26] scores, which range from 0 to 1, were calculated to assess the probability of pathogenicity, as defined by the program. Additionally, PolyPhen-2 [27] (http://genetics.bwh.harvard.edu/pph2/bgi.shtml) analysis was performed to predict the possible impact of amino acid substitutions on protein structure and function, with scores ranging from 0 (benign) to 1 (probably damaging).

### Protein structure prediction and analysis

The JAK2 structure was predicted using AlphaFold3 (https://alphafoldserver.com). Structural analysis and visualization were performed using PyMOL (version 2.4.1).

### Protein structure prediction and analysis

The JAK2 structure was predicted using AlphaFold3 (https://alphafoldserver.com). Structural analysis and visualization were performed using PyMOL (version 2.4.1).

## Results

### Characterization and functional assessment of JAK/STAT missense variants in an alpine cohort

**Fig. 1:**
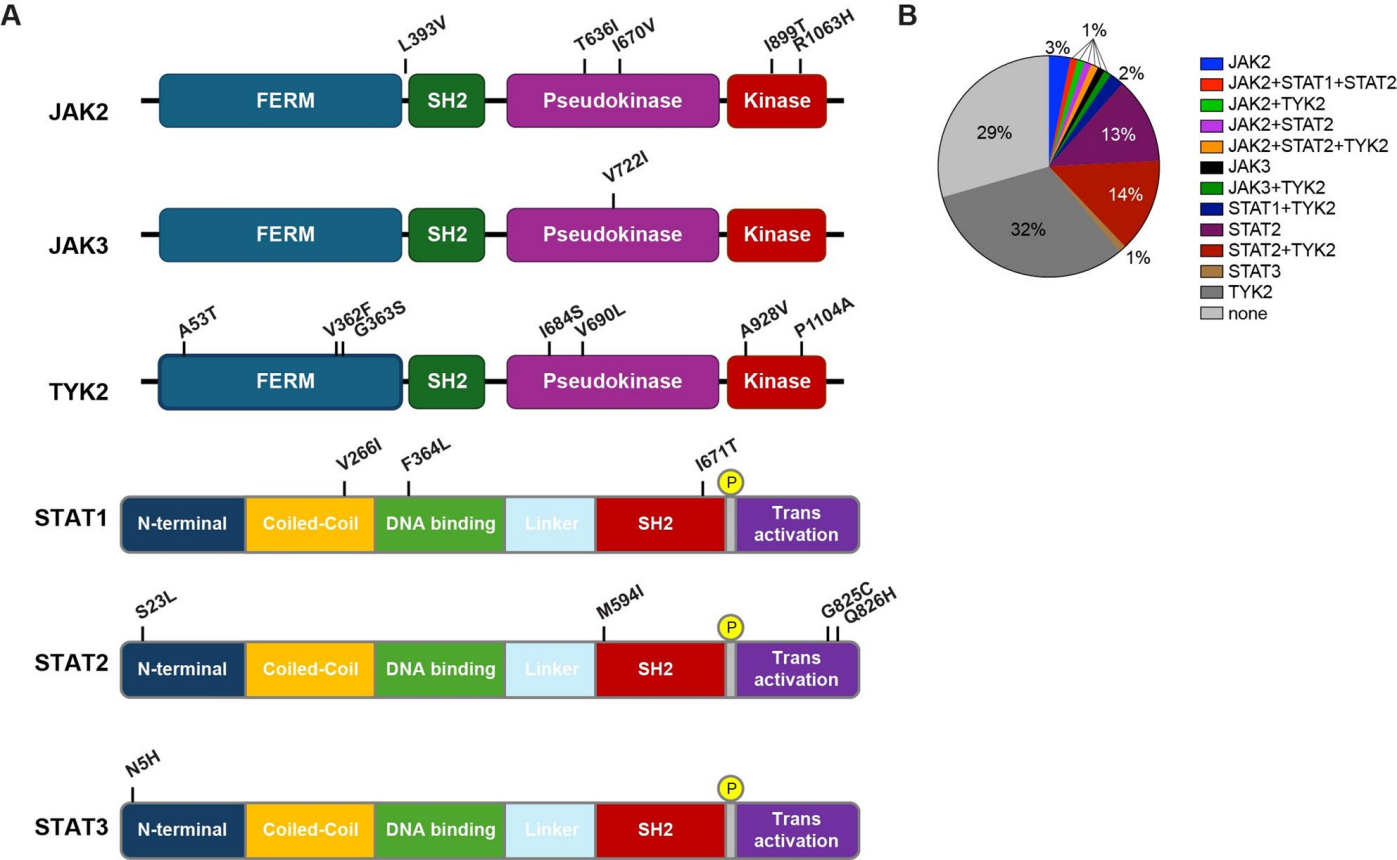
JAK/STAT variants identified. (**A**) Linear domain structures of JAK/STAT/TYK2 proteins and the position of variants identified in the Alpine cohort. (**B**) Distribution of individual and combinatorial variants observed within the cohort

Here we analyzed missense variants in the JAK/STAT signaling pathway genes in 98 individuals from an Austrian alpine community and associate them with immune transcriptomes obtained 4–6 weeks after COVID-19 infection [8]. First, we identified single nucleotide variants (SNVs) in protein coding sequences using RNA-seq data from all 98 study subjects. Altogether 21 different distinct variants were identified in the *JAK2*, *JAK3* and *TYK2* genes as well as in the *STAT1*,* STAT2* and S*TAT3* genes (Table 1; Fig. 1A). Whole genome sequencing (WGS) was used to validate variants in selected individuals. The allele frequencies (AF) of these germline variants, based on the gnomAD and AllofUs databases, ranged from approximately 10^− 6^ for the very rare JAK2^T636I^, JAK2^I670V^, STAT1^I671T^ and STAT2^S23L^ variants to approximately 10^− 1^ for the common TYK2^V362F^ variant. Variants were identified in the receptor interacting Band 4.1, Ezrin, Radixin, Moesin (FERM) domain (TYK2), the regulatory pseudokinase domain (JAK2, JAK3 and TYK2) and the kinase domain (JAK2 and TYK2). STAT variants were present in the N-terminal region (STAT2 and STAT3), the coiled-coil and DNA binding domain (STAT1), the Src-homology 2 (SH2) domain (STAT1 and STAT2) and the transactivation domain of STAT2 (Fig. 1A).

**Table 1:** JAK and STAT variants identified in study subjects

Gene	AA substitution	rsID	gnomAD		All of Us		COSMIC
			Allele counts	Allele frequency	Allele counts	Allele frequency	Patient #
JAK2	L393V	rs2230723	10,394	6.47E-03	10,442	1.26E-02	4
	T636I	rs1819381844	2	1.43E-06	4	5.00E-06	0
	I670V	rs771779649	57	3.54E-05	20	2.40E-05	0
	I899T	rs200282557	170	1.06E-04	140	1.69E-04	1
	R1063H	rs41316003	8630	5.39E-03	3635	4.38E-03	9
JAK3	V722I	rs3213409	15,743	9.75E-03	7721	9.31E-03	59
TYK2	A53T	rs55762744	14,980	9.29E-03	6143	7.41E-03	0
	V362F	rs2304256	446,002	2.76E-01	200,826	2.42E-01	17
	G363S	rs2304255	113,510	7.03E-02	47,106	5.68E-02	1
	I684S	rs12720356	128,313	7.97E-02	52,376	6.31E-02	5
	V690L	-	-	-	-	-	1
	A928V	rs35018800	10,241	6.35E-03	4117	4.96E-03	6
	P1104A	rs34536443	59,598	3.71E-02	24,207	2.92E-02	3
STAT1	V266I	rs41473544	5072	3.14E-03	1938	2.34E-03	0
	F364L	rs759722579	198	1.27E-04	98	1.18E-04	0
	I671T	rs747656964	60	3.72E-05	21	2.50E-05	1
STAT2	S23L	-	7	4.34E-06	4	5.00E-06	0
	M594I	rs2066807	93,983	5.82E-02	41,434	4.99E-02	5
	G825C	rs61754170	29,650	1.84E-02	10,224	1.23E-02	1
	Q826H	rs2229363	14,384	8.91E-03	7259	8.75E-03	0
STAT3	N5H	-	-	-	-	-	0

To determine if any of the JAK/STAT variants had previously been reported in the context of disease, we employed the COSMIC and NIH ClinVar databases. To explore the potential of altered function of the JAK/STAT variants based on structural features, we employed several in silico prediction tools (Table 1, Table S3). Out of the 21 germline variants, 13 were also found in the COSMIC database, suggesting a gain-of-function (GOF) activity of these variants. The most prominent variants in COSMIC was the JAK3^V722I^, a known GOF variant [28, 29], with 59 entries. Among the very rare germline variants, only JAK2^I899T^ and TYK2^V690L^ were found in the COSMIC database. However, both are considered variants of unknown significance (VUS). ClinVar categorized 11 variants as benign or likely benign, two as uncertain significance and three were associated with ‘conflicting classifications’ (Table S3). There were no entries in ClinVar for the very rare variants. AlphaMissense [25], a state-of-the-art computational tool, predicted pathogenic scores (0.56–1.56) for TYK2^I684S^, TYK2^A928V^, TYK2^P1104A^ and STAT1^F364L^. In contrast, PolyPhen2 [27] analysis predicted eight variants as deleterious, including TYK2^I684S^, TYK2^A928V^ and TYK2^P1104A^, which also had the highest pathogenic score in AlphaMissense. The REVEL (Rare Exome Variant Ensemble Learner) [26] score predicted five alleles as deleterious, including JAK2^I899T^, TYK2^A928V^ and TYK2^P1104A^, which also scored high in all three prediction tools. Not surprisingly, different tools provided a range of pathophysiological predictions for the mutants, creating a challenge to identify their i*n vivo* function in regulating basal immune transcriptomes and innate immune responses following viral infection.

Twenty-one JAK/STAT variants were identified. JAK2^R1063H^ and JAK3^V722I^ have previously been shown to have elevated activity [28–31]. The TYK2^A928V^ and TYK2^P1104A^ variants are reported to have impaired catalytic activity, leading to reduced autophosphorylation and diminished downstream signaling in response to cytokines, suggesting that they might be hypomorphic alleles, resulting in partial loss of function [32]. Missense variants were also identified in the *STAT1*,* STAT2* and *STAT3* genes (Fig. 1A; Table 1). Except for the STAT1^V266I^ variant, which had no impact [33], the other variants have not been studied biochemically.

Out of the 98 study subjects, only 31 did not carry any JAK/STAT missense variants (Fig. 1B). Allele frequency of the JAK/STAT variants in the gnomAD and *All of Us* databases as well as the number of patients reported in the COSMIC database are presented in Table 1. One key finding of the study was the common occurrence of two or more JAK/STAT variants in one third of the study participants, and the co-occurrence of different variants (Table S2). Out of the nine individuals carrying JAK2 or JAK3 variants, five carried additional variants in STAT1, STAT2 or TYK2. In general, TYK2 variants were the most common ones in this cohort, with 48 individuals carrying one or more TYK2 variants. Among the 31 individuals carrying STAT variants, 18 carried additional variants, mainly in TYK2. Interestingly, seven out of 15 individuals positive for the common STAT2^M594I^ variant did not carry another JAK/STAT variant. The complexity of co-existing JAK/STAT variants within individuals complicates the interpretation of biological data as the combinatorial epistatic interactions are unknown.

### Linking JAK/STAT variants to enhanced immune transcriptomes

**Fig. 2:**
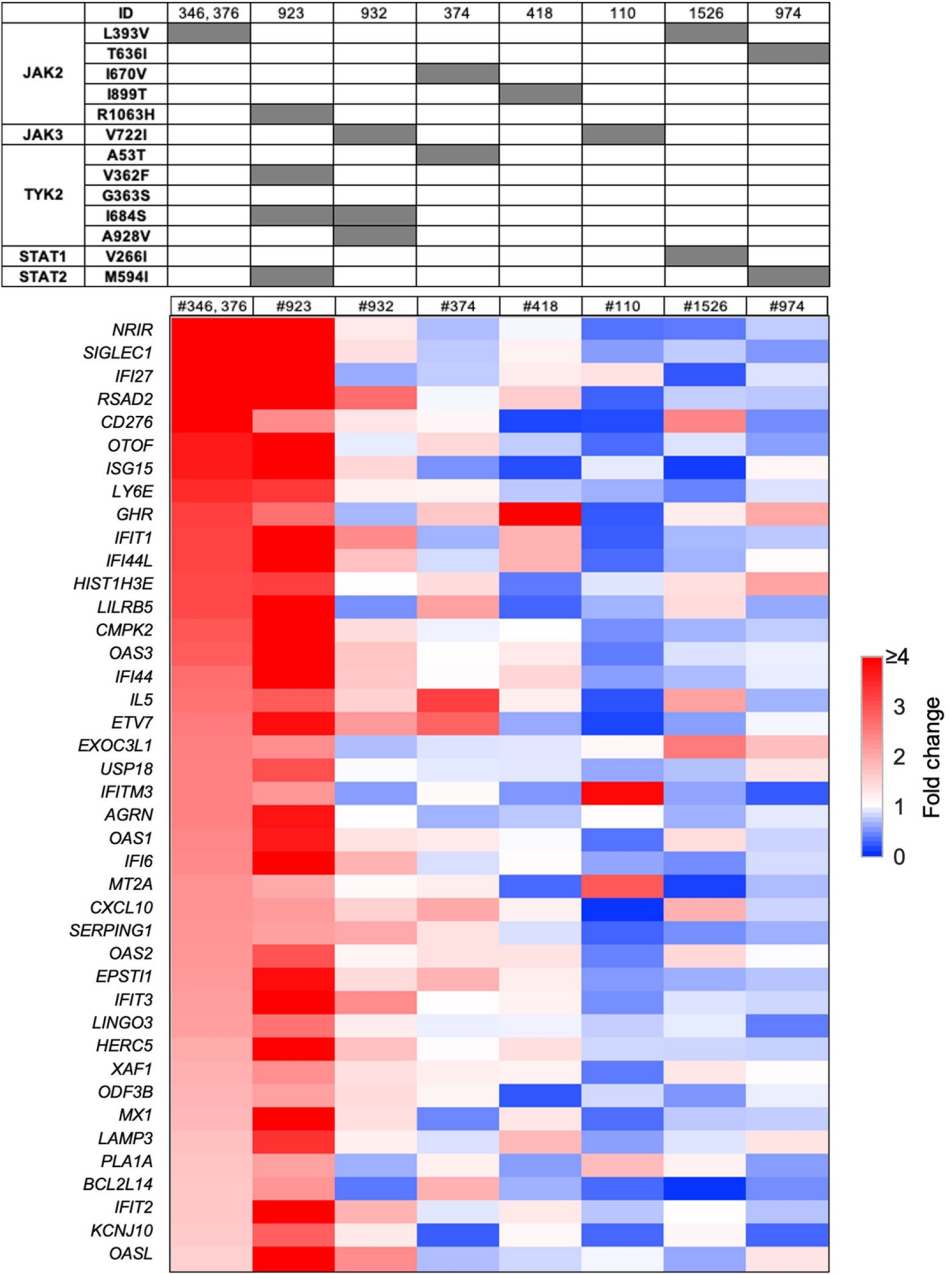
Transcriptional response of JAK/STAT target genes in individuals with JAK variants. Heatmap depicting the fold change in expression of interferon-stimulated genes compared to individuals without JAK/STAT variants

To explore the potential impact of the JAK/STAT variants on the baseline immune transcriptome in a real-world setting, we directly investigated the immune transcriptomes from individuals carrying one or more variants. For this, we used a reference set of 41 genes known to be activated through the JAK/STAT pathways and specifically by interferons (Table S4). This gene set includes interferon-induced genes that encode antiviral proteins (OAS proteins), chemokine families (CXCL), interferon Induced proteins with tetratricopeptide repeats (IFITs) known to confer immunity against viral infections and transcription factors (XAF1, ETV7). First, we compared the transcriptomes of the nine individuals carrying JAK2 and JAK3 variants with the transcriptomes of 27 individuals from the same community not carrying any JAK/STAT variant. Elevated expression of the immune gene set was observed in the two individuals carrying the JAK2^L393V^ variant and in the individual carrying the JAK2^R1063H^ variant (Fig. 2; Table S4). While the two individuals carrying the JAK2^L393V^ variant did not carry another JAK/STAT variant the individual #923 with the JAK2^R1063H^ variant also carried the TYK2^I684S^, TYK2^V362F^ and STAT2^M594I^ variants. While the STAT2^M594I^ variant is of unknown significance (VUS) the TYK2^I684S^ variant has been suggested to be protective against Rheumatoid Arthritis (RA) and Systemic Lupus Erythematosus (SLE) [34] with a potential to contribute to the elevated transcriptome. However, none of the study subjects carried exclusively the TYK2^I684S^ variant and the co-occurrence of up to four different JAK/STAT variants in some individuals would yield a more complex response, going beyond the function of an individual variant and possibly masking the biological effects of some variants.

Of note, individual #1526 who carried the JAK2^L393V^ variant in combination with the STAT1^V266I^ variant, did not display an enhanced innate immune transcriptome suggesting that either the STAT1 variant does not fully convey the JAK2 activity or additional variants in the extended interferon pathway contributed to the response. Two individuals carried the know activating variant JAK2^V722I^ but only individual #932 showed limited activation of an Interferon-Stimulated Genes (ISG)-type signature. This female individual also carries a mutation in the *NEMO* gene (Inhibitor of Nuclear Factor Kappa B Kinase Regulatory Subunit Gamma, *IKBKG***)**. *IKBKG* plays a central role in immune regulation, primarily by controlling the NF-κB signaling pathway, which is essential for innate and adaptive immune responses. No significant elevated ISG expression was observed in individuals carrying the other JAK2 variants, either alone or in combination with TYK2 variants.

**Fig. 3:**
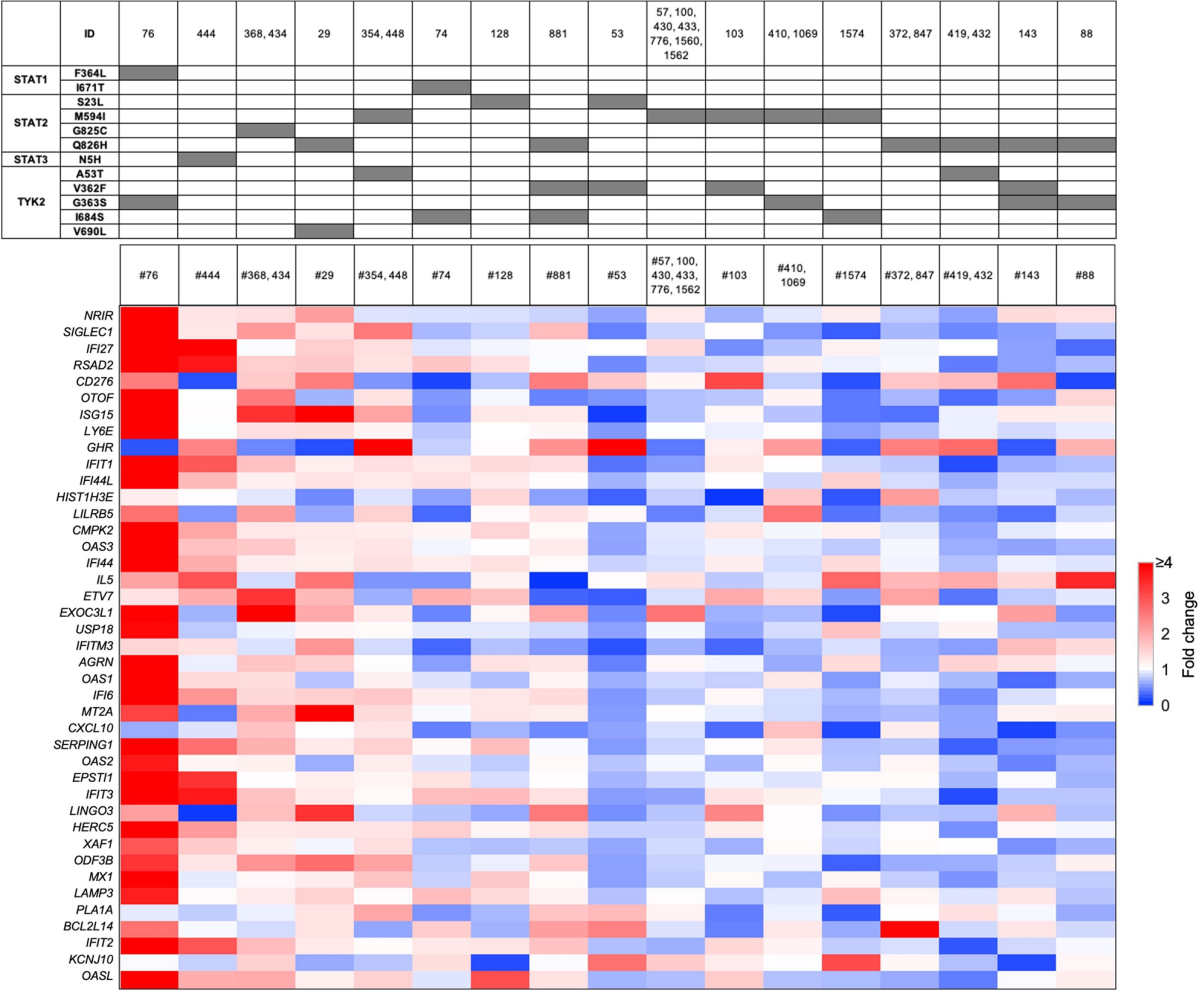
Immune transcriptome of individuals carrying STAT variants. Heatmap showing the relative expression levels of interferon-stimulated immune genes in individuals with STAT variants and without JAK/STAT variants

Among the STAT variants, only the individual carrying the rare STAT1^F364L^ variant displayed an elevated immune transcriptome, with induction of up to 27-fold in some genes (Fig. 3; Table S4). No impact of the more common STAT2 variants M594I and Q826C was observed, either by themselves or in combination with TYK2 variants.

**Fig. 4:**
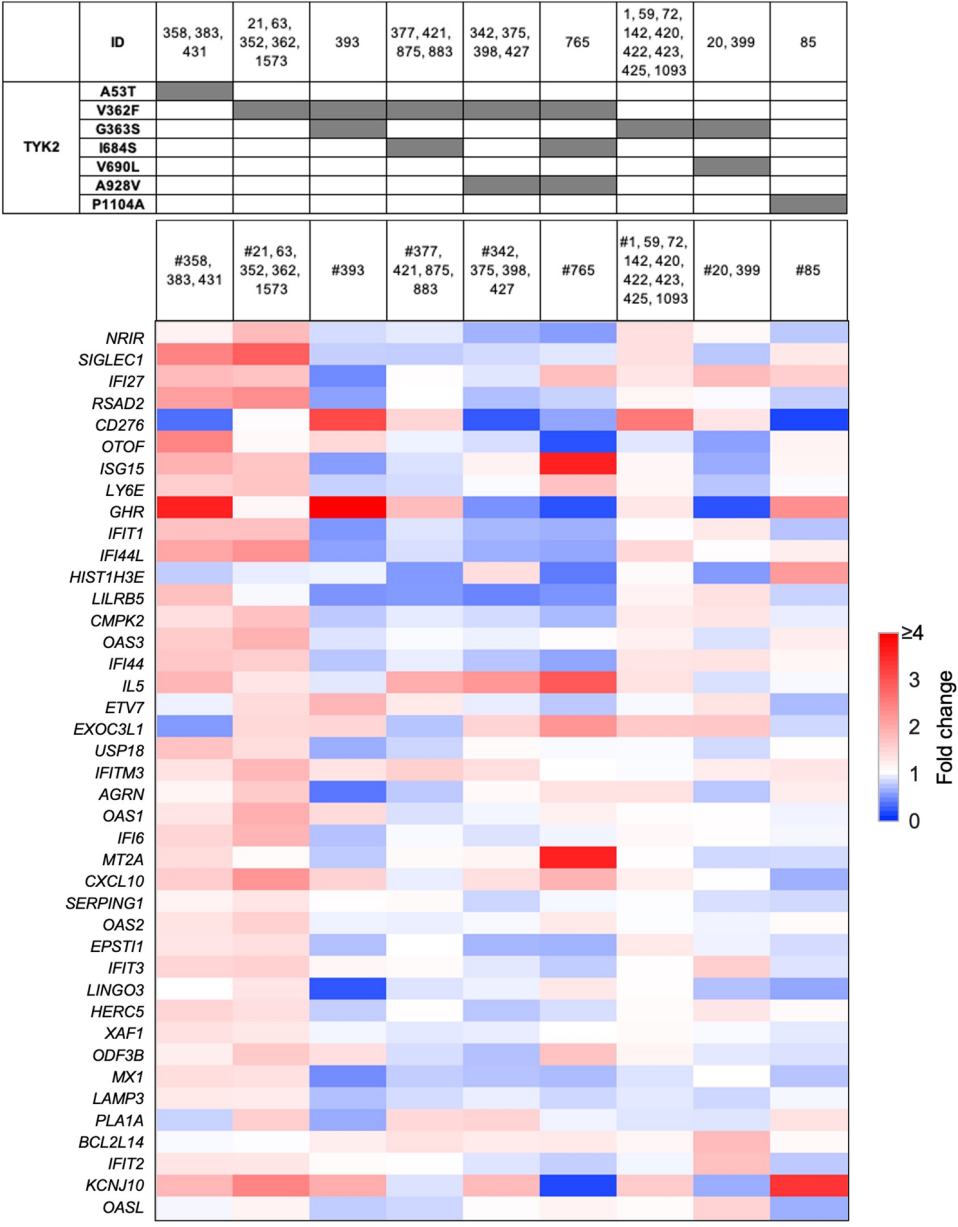
Transcriptional response of JAK/STAT target genes in individuals with TYK2 variants. Heatmap displaying fold changes in interferon-stimulated gene expression associated with TYK2 variants

The majority of individuals carrying TYK2 variants did not demonstrate an enhanced immune signature, either exclusively or combined with different STAT variants (Figs. 3 and 4). However, enhanced immune signatures were observed in the three individuals carrying exclusively the TYK2^A53T^ variant and the five individuals carrying exclusively the TYK^V362F^ variant (Fig. 4) as well as individual 76 who carries a combination of the common TYK2^G363S^ and the rare STAT1^F364L^ variant (Fig. 3).

### Structural consequences of variants

**Fig. 5:**
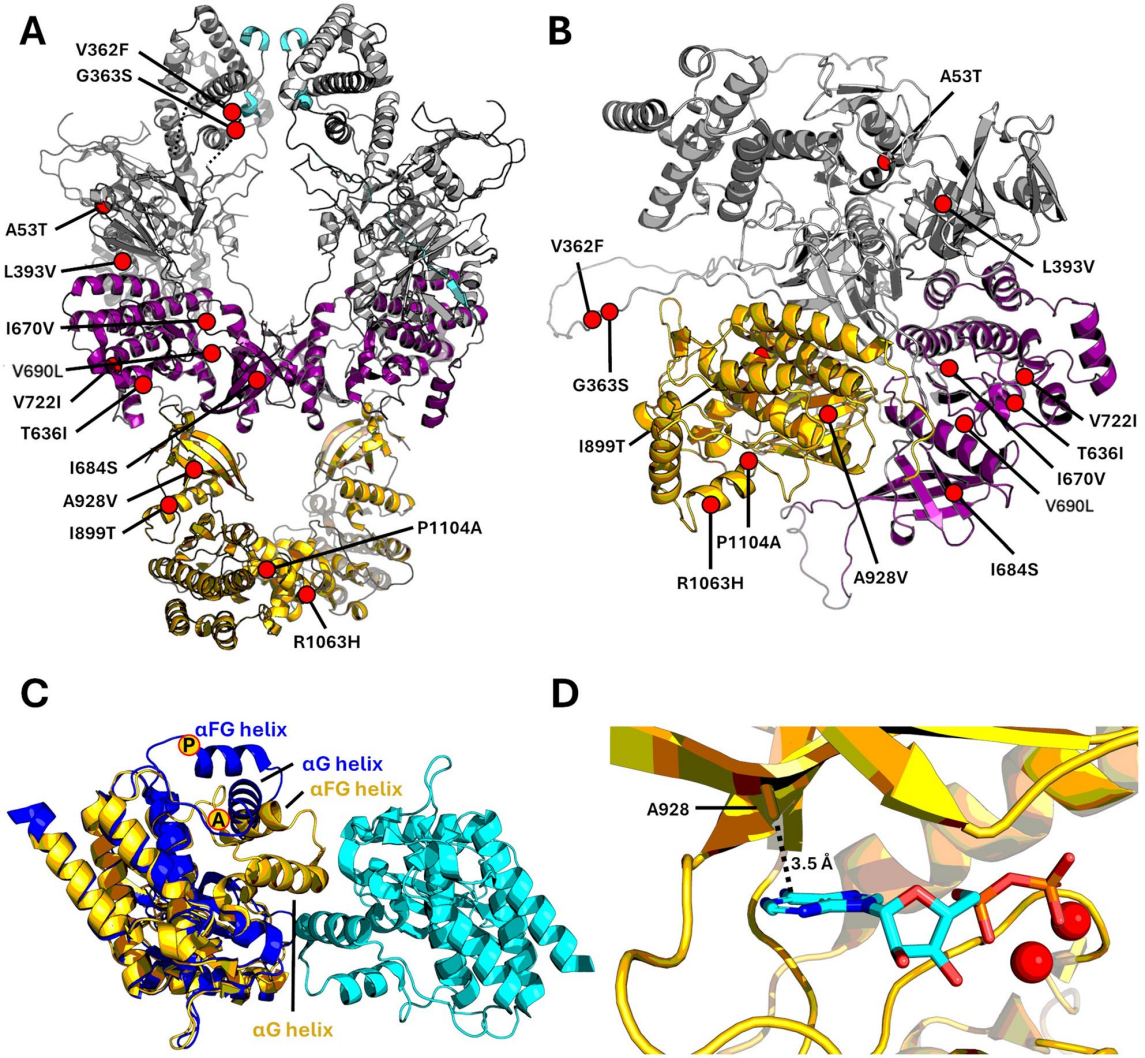
Structural analysis. (**A**-**B**) The identified variants mapped in a cryo-EM structure of a JAK1 dimer (**A**) and AF3 prediction of TYK2 monomer (**B**). FERM-SH2 domains are shown in gray, pseudokinase domains in purple and kinase domains in gold. (**C**) TYK2 P1104A homolog (JAK2 P1057A, PDB code 5HEZ, in blue) was superimposed with the kinase domains (in gold and cyan) of a trans-activated JAK1 dimer (PDB code 8EWY). ‘P’ and ‘A’ mark the positions of the wild-type and mutated residues, respectively. The mutation disrupts the kinase-kinase interface, especially affecting the position of the central αG-helix. (**D**) TYK2 kinase domain in complex with Mg-ADP (PDB code 4GVJ) shows the proximity of Ala928 to the adenine base of the nucleotide. Mutation of Alanine to Valine would likely lead to steric clash between the nucleotide and the mutated residue

Next, we used structural analysis and AlphaFold3 [35, 36] to predict the structural impacts of the JAK and STAT variants. We mapped all the JAK variants into an active JAK dimer structure (murine JAK1^V685F^ mutant) [37] and a monomeric JAK structure from AF3 prediction possibly representing an autoinhibited JAK conformation (Fig. 5A, B). Utilizing these two JAK conformations and available crystal structures, we analyzed the potential structural effects of the identified variants.

JAK2^L393V^ located in the FERM-SH2 domain has been reported to be weakly hypersensitive to erythropoietin (EPO) stimulation [30]. The residue is in a hydrophobic pocket, and the variant could affect the packing of the surrounding residues although the overall effect might be predicted to more of moderative rather than defining nature. JAK2^T636I^ and JAK2^I670V^ are within the pseudokinase domain. Based on the JAK1 cryo-EM structure, T636I is located at the pseudokinase-kinase interface, and thus has a potential to modulate the trans-activated conformation. I670V resides in the catalytic loop and has been found to be associated with a myelodysplastic phenotype [38]. However, the Ile to Val variant is very conservative and does not have any evident structural effects. JAK2 kinase domain mutant I899T resides in the regulatory αC-helix but the isoleucine is not engaged in any specific interactions, and the variant appears to be mostly benign. The other JAK2 kinase domain variant, R1063H, activates JAK2 signaling when cooccurring with pathogenic V617F. The variant resides close to the kinase-kinase interface in the transactivation pose and could thus modulate JAK2 activity.

JAK3^V722I^ has been identified as an activating variant in natural killer T-cell lymphoma and acute megakaryoblastic leukemia [28, 29]. This variant itself is conservative. Isoleucine slightly increases the size of the residue but both amino acids are hydrophobic. The variant is located at the C-lobe of the pseudokinase domain, and the structural models do not directly explain the activating phenotype of this mutant.

TYK2 variants A53T, V362F, and G363S are located at the FERM domain. A53T has been identified as a risk allele in multiple sclerosis (MS) [39]. The residue is located in a pocket lined by histidine, proline and leucine (pdb code 4PO6 [40]) and variant of alanine to polar threonine will likely lead to clashes and disruption of the structure. Based on being an MS risk allele, the variant is likely GOF. TYK2^V362F^ and TYK2^G363S^ are located in a long flexible loop, which is not visible in TYK2 crystal structures or in JAK1 cryo-EM structures. In AF3 predictions, the loop is fully disordered and solvent exposed. The TYK2^G363S^ variant was tested for the magnitude of autophosphorylation in response to interferon (IFN)-α following transfection of an expression vector into TYK2-deficient U1A cells but the autophosphorylation response was no different than found with the transfected wild-type vector [41]. Study of the TYK2^V362F^ variant showed that while the amino acid substitution does not alter TYK2 catalytic activity, the nucleotide change does alter exon splicing, promoting exon 8 inclusion in association with mildly higher mRNA expression levels in blood [42]. TYK2^I684S^ has been described as having impaired catalytic activity and is considered a psoriasis-protective variant [43, 44]. The altered amino acid structure of TYK2^I684S^ is located close to the dimerization interface with JAK1 based on cryo-EM structural analysis. It is possible that TYK2^I684S^ might exhibit an altered dimerization pattern with JAK1. TYK2^P1104A^ is described as a protective variant for autoimmune diseases [45] but one that can increase susceptibility to tuberculosis infection [32]. This catalytically impaired mutant [43] is homologous to Rs34536443, which has been shown to lead to drastic local structural changes [46]. Structural analysis was used to evaluate the impact of the proline to alanine change. This was done by superposing the TYK2 P1104A homolog (JAK2 P1057A, PDB code 5HEZ) structure into an active JAK1 dimer structure (PDB code 8EWY). The change leads to a significant rearrangement of αFG and αG helices in the kinase domain and can be seen to disrupt the kinase-kinase interface in the transactivated state (Fig. 5C). This could explain the catalytically impaired phenotype. The TYK2^V690L^ variant lies at the hinge of the pseudokinase domain and makes a direct interaction with the adenine of the bound nucleotide via the main chain amide [47]. The side chain of the valine points towards two β-strands (β7 and β8). This could affect hinge conformation and nucleotide binding to the ATP pocket. TYK2^A928V^ lies at a β-strand (β3) lining the ATP-pocket with the side chain directly pointing towards the adenine base of the bound nucleotide. Changing the alanine to a bulkier valine very likely requires structural rearrangements in the pocket to accommodate ATP and could translate into changes in catalytic activity (Fig. 5D).

Structural analysis of the STAT variants did not offer any clues to how they might disrupt STAT function. STAT2^G825C^ is located outside the core STAT fragment utilized in crystallographic studies and this region is also fully disordered in the AF3 model of STAT2. The STAT2^M594I^ variant in the SH2 domain and is also solvent exposed. Because oxidation of the methionine has been found to abolish IFN or virus-triggered activation of STAT2 [48], it is possible that this variant disrupts redox regulation of STAT2. STAT1^V266I^ is located in the coiled coil domain with the side chain facing the center of the coiled coil bundle. The valine to isoleucine change could disrupt the bundle structure by introducing a larger side chain, but, to date, this variant has not been observed to influence STAT1 phosphorylation [33]. The phenylalanine to leucine change of STAT1^F364L^ lies within the DNA-binding domain however, structural analyses show that the specific region with the amino acid change does not directly interact with DNA and it was not classified as a GOF variant based on an alanine-scanning mutagenesis study [49]. STAT1^I671T^ is located in the SH2 domain, and its side chain is solvent-exposed without any predicted structural consequences. The STAT2^S23L^ and STAT3^N5S^ variants alter surface-exposed residues in the N-terminal domain and similarly there are no clear structural effect for these changes available in experimental structures. STAT2^Q826H^ is in the C-terminal transactivation domain (TAD). Little structural information exists on STAT TADs and based on an NMR structure [50], the residue is facing the solvent and not involved in specific interactions. Other STAT variants (STAT1^V266I^, F364L and I671T, STAT2^S23L^ and STAT2^Q826H^ and STAT3^N5S^) are either located on missing domains (N) or at the surface thus providing little or no information on their function.

## Discussion

Here we identify 21 variants in JAK/STAT components and find three variants (JAK2^L393V^, JAK2^R1063H^, STAT1^F364L^) associated with an elevated baseline immune transcriptome. Individuals carrying the JAK2^L393V^, JAK2^R1063H^ or the STAT1^F364L^ variant display increased levels of interferon-induced genes linked to antiviral programs (OAS and IFIT proteins), chemokine families (CXCL), interferon Induced proteins with tetratricopeptide repeats (IFITs) and transcription factors (XAF1, ETV7). Both JAK2^L393V^ and JAK2^R1063H^ appear to be GOF variants [31, 51]. The JAK2^R1063H^ variant has been identified in hereditary erythrocytosis and shown to augment JAK2 activity and enhance STAT5 phosphorylation and transcriptional activity [51] as well as being linked to thrombosis and leukemic transformation in mice [31]. The JAK2^L393V^ increases EPO responsiveness [30]. The most profound increase of key interferon-induced genes, including a more than 10-fold induction of several key genes controlling the innate immune system, was seen in the individual carrying the STAT1^F364L^ variant, located in the DNA binding domain (DBD). The DBD has been recognized as a hotspot for STAT1 GOF variants [52–54] but this specific mutation has not previously been associated with enhanced activity. JAK3^V722I^, a known activating variant was found in two individuals. An elevated ISP signature was only found in the female subject (#932) who also carries the NEMO variant.

The assignment of a biological response to a single missense variant is challenged by the complexity and existence of co-existing variants in the extended JAK/STAT signaling pathway including receptors and downstream regulators. The most profound induction of interferon-regulated genes was observed in the individual carrying the STAT1^F364L^ and TYK2^G363S^ variants. While the activity of the TYK2 variant is considered identical to wild-type TYK2, a unique interaction with the STAT1 variant cannot be excluded. WGS of this individual revealed additional rare and common missense variants in the extended JAK/STAT pathway, including the transcription factors *IRF3* and *IRF7*, *IFIH1* and *DHX58* (*LGP2*), all of which are crucial to the body’s innate immune system.

Activation of the innate immune response relies on multiple components, such as ligands, receptors, JAKs, STATs and other downstream transcription factors and negative regulators. Activation of cytokines signaling requires pairing of two JAK kinases. Presently our knowledge of the structural basis of cytokine signaling and its link to pathogenesis is predominantly based on JAK2 and its homodimeric activation in hormone receptors such as the EPO Receptor. Most cytokine receptors acting in the innate immune response bind two different JAKs after cytokine stimulation: IFN-α with JAK1/TYK2, IFN-g with JAK1-JAK2 and Interleukin (IL)−12 with TYK2/JAK2. The mechanisms and structural basis of heterodimer JAK activation is still elusive and will be critical for understanding the molecular basis of the various variants and their interplay in different signaling components. One striking finding of this study was the lack of JAK1 variants in the cohort. JAK1 is used by almost 30 cytokine receptors and is particularly important in inflammatory responses and it is possible that JAK1 variants are too detrimental. Similarly, variants were found in STAT1, 2 and 3 but not in STATs 4–6. This complexity of variants and the endless possibilities of epistasis [55] challenges the notion that specific immune responses within the normal range can be linked to individual variants. While germline variants in JAK2 and the erythropoietin receptor [56] have been linked unequivocally to erythrocytosis, future studies will need to focus increasingly on the combinations of variants found in normal populations and in families differentially afflicted by a specific variant, such as the Mäntyranta variant [56]. There even could be differences between geographic areas. For example, the STAT2^M594I^ and TYK2^G363S^ variants have been linked to increased susceptibility to SARS-CoV-2 infection in a Moroccan population [57] but we did not observe that here.

Links between the JAK/STAT genetic variants and autoinflammatory disease (AIDs) have been drawn and JAK/STAT inhibitors show particular promise in treating interferonopathies [58]. Dysregulation of the JAK/STAT pathway due to loss or gain of function variants has been associated with a range of clinical phenotypes that are increasingly being understood to be more nuanced than the simple development of autoimmunity, immunodeficiency or malignancy [59]. Significantly, in this population, the individual variants and variant combinations observed were not associated with known changes in infection susceptibility or increased risk of autoimmune diseases, allergy, cancer or blood-related disorders in any statistically significant manner. Because the study is a single timepoint and study subjects exhibited a wide range of ages, one also cannot determine who might develop immunological or malignant disease with age. The small size of the present study produces weak statistical power. Knowledge about how the various variants might impact immune responses would be aided by larger family studies that could follow individuals over time, including taking note of how life events and environmental exposures might influence expression of disease. With the low allele frequency of some of the variants and the complexity of co-segregating variants this type of study would require a stable investment over many years. Additional approaches to understanding the significance of rare variants would be to employ a network-based modeling approach once sufficient numbers of datapoints become available [60]. In the future there may be sufficient resources to perform more wide-ranging functional studies using primary cells from the individuals carrying the various variant combinations with experimental exposure to specific cytokines. The three variants JAK2^L393V^, JAK2^R1063H^ and STAT1^F364L^ associated here with elevated levels of specific immune regulated genes would be of particular interest for further study.

## Limitations of the study

A key limitation of this study is that many of the variants are represented by single individuals, reflecting the extreme rarity of these protein-coding variants, which occur at frequencies below 1 in 10,000 in the general population. This small sample size produces weak statistical power. Due to the rarity of both the individual variants and the variant combinations, it was not feasible to assemble larger cohorts of individuals with the same variant patterns within the scope of the study. This is a key study restriction.

## Supplementary Information

Supplementary Material 1.

Supplementary Material 2.

Supplementary Material 3.

Supplementary Material 4.

## Data Availability

The RNA-seq data used in this study were obtained from the Gene Expression Omnibus (GEO), accession number GSE162562 [8]. The GEO data downloaded for this study was originally obtained from an investigation of the transcriptional response to past COVID infection. The whole genome sequencing data generated in this study has not been deposited in a public database due to privacy concerns. Researchers interested in accessing the dataset may contact the authors directly.
