# Supplementary material for: Investigation of the transcriptional impact of rare germline JAK/STAT variants found in a Tyrolean alpine community
Source: BMC Genomics. 2025 Dec 1;27:8. doi: 10.1186/s12864-025-12307-0 (PMC12771743; doi:10.1186/s12864-025-12307-0)
Supplement: Supplementary file 2 — Supplementary Material 2. [file 12864_2025_12307_MOESM2_ESM.docx]

**Table S2. JAK/STAT variant patterns and reported immunologically-related disease, infection or cancer, allergies and family household relationships.**


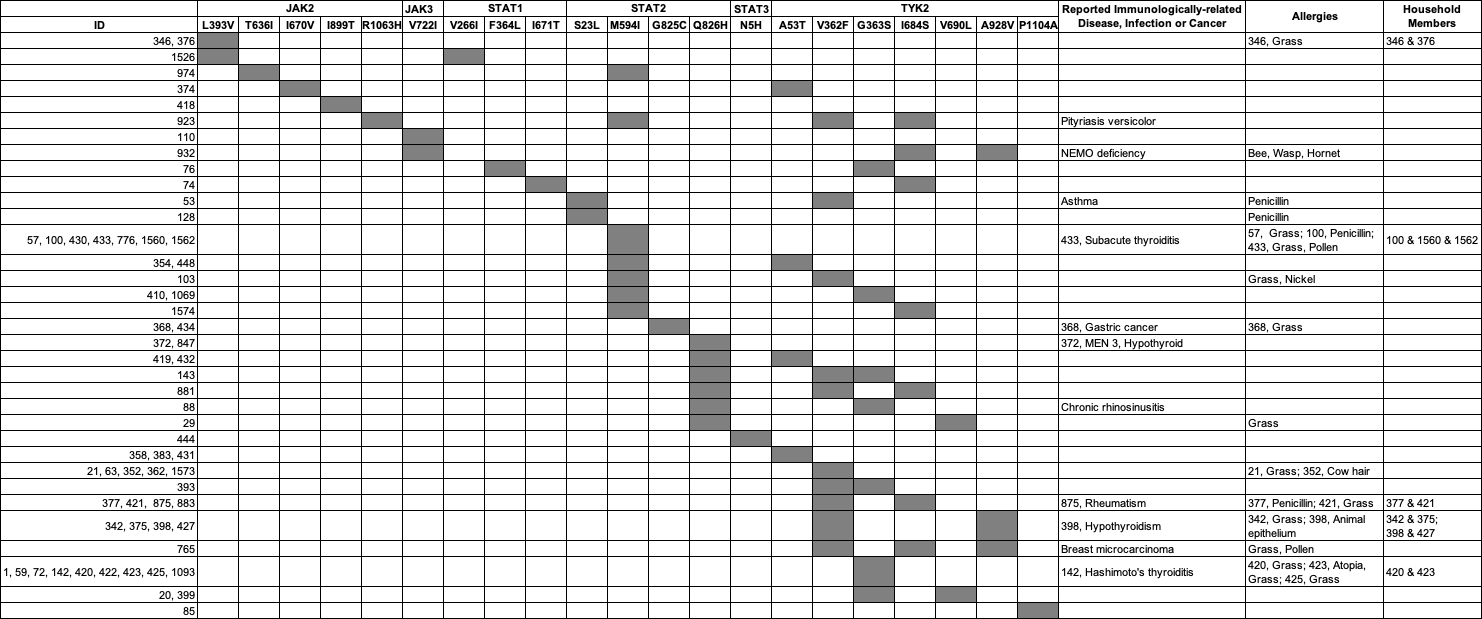


*Nemo: Nuclear factor-kappa B Essential Modulator (IKBKG exon4_10del mutation)

*MEN: Multiple Endocrine neoplasia
