# Supplementary material for: Investigation of the transcriptional impact of rare germline JAK/STAT variants found in a Tyrolean alpine community
Source: BMC Genomics. 2025 Dec 1;27:8. doi: 10.1186/s12864-025-12307-0 (PMC12771743; doi:10.1186/s12864-025-12307-0)
Supplement: Supplementary file 3 — Supplementary Material 3. [file 12864_2025_12307_MOESM3_ESM.docx]

**Table S3. Potential clinical pathogenicity of JAK and STAT variants.**

| **Gene** | **AA substitution** | **rsID** | **ClinVar** | **In silico pathogenicity score** | | | **References (PMID)** |
| --- | --- | --- | --- | --- | --- | --- | --- |
|  |  |  | **Clinical significance** | **AlphaMissense** | **PolyPhen2** | **REVEL** |  |
| **JAK2** | L393V | rs2230723 | Conflicting classifications | 0.080 | 0.044 | 0.091 | 27647865 |
|  | T636I | rs1819381844 | - | 0.118 | 0.974 | 0.136 | - |
|  | I670V | rs771779649 | - | 0.148 | 0.077 | 0.669 | - |
|  | I899T | rs200282557 | Uncertain significance | 0.340 | 0.994 | 0.925 | - |
|  | R1063H | rs41316003 | Benign/Likely benign | 0.109 | 0.013 | 0.276 | 30377194  [27389715](https://pubmed.ncbi.nlm.nih.gov/27389715/) |
| **JAK3** | V722I | rs3213409 | Benign | 0.071 | 0.003 | 0.156 | 38244120  16843266 |
| **TYK2** | A53T | rs55762744 | Benign | 0.158 | 0.922 | 0.458 | - |
|  | V362F | rs2304256 | Benign | 0.083 | 0.021 | 0.053 | 31961910 |
|  | G363S | rs2304255 | Benign | 0.075 | 0.000 | 0.027 | 18270328 |
|  | I684S | rs12720356 | Benign | 0.586 | 0.968 | 0.343 | 23359498  29728633  [30578352](https://pubmed.ncbi.nlm.nih.gov/30578352/) |
|  | V690L | - | - | 0.249 | 0.126 | 0.279 | - |
|  | A928V | rs35018800 | Benign/Likely benign | 0.928 | 1.000 | 0.817 | 31961910  [36094518](https://pubmed.ncbi.nlm.nih.gov/36094518/) |
|  | P1104A | rs34536443 | Benign/Likely benign | 0.829 | 1.000 | 0.586 | 31961910  23359498  [30578352](https://pubmed.ncbi.nlm.nih.gov/30578352/)  [36094518](https://pubmed.ncbi.nlm.nih.gov/36094518/) |
| **STAT1** | V266I | rs41473544 | Conflicting classifications | 0.079 | 0.012 | 0.287 | 26604104 |
|  | F364L | rs759722579 | Conflicting classifications | 0.920 | 0.033 | 0.302 | - |
|  | I671T | rs747656964 | Uncertain significance | 0.075 | 0.555 | 0.611 | - |
| **STAT2** | S23L | - | - | 0.082 | 0.015 | 0.123 | - |
|  | M594I | rs2066807 | Benign | 0.084 | 0.000 | 0.36 | - |
|  | G825C | rs61754170 | Benign | 0.086 | 0.013 | 0.013 | - |
|  | Q826H | rs2229363 | Benign/Likely benign | 0.115 | 0.007 | 0.082 | - |
| **STAT3** | N5H | - | - | 0.150 | 0.986 | - | - |
